# Homozygous mutation in the *Neurofascin* gene affecting the glial isoform of Neurofascin causes severe neurodevelopment disorder with hypotonia, amimia and areflexia

**DOI:** 10.1093/hmg/ddy277

**Published:** 2018-08-13

**Authors:** Robert Smigiel, Diane L Sherman, Małgorzata Rydzanicz, Anna Walczak, Dorota Mikolajkow, Barbara Krolak-Olejnik, Joanna Kosińska, Piotr Gasperowicz, Anna Biernacka, Piotr Stawinski, Malgorzata Marciniak, Witalij Andrzejewski, Maria Boczar, Paweł Krajewski, Maria M Sasiadek, Peter J Brophy, Rafal Ploski

**Affiliations:** 1 Department of Pediatrics and Rare Disorders, Wroclaw Medical University, Wroclaw 51-618, Poland; 2 Department of Medical Genetics, Medical University of Warsaw, Warsaw 02-106, Poland; 3 Department of Neonatology, Wroclaw Medical University, Wroclaw 55-556, Poland; 4 Department of Neonatology, Provincial Specialist Hospital, Wroclaw 51-124, Poland; 5 Hospice for Children and Adults, Lodz 91-496, Poland; 6 Department of Genetics, Wroclaw Medical University, Wroclaw 50-367, Poland; 7 Centre for Discovery Brain Sciences, University of Edinburgh, Edinburgh EH16 4SB, UK; 8 Clinics of Surgery of Children and Adolescents, Institute of Mother and Child, Warsaw 01-211, Poland; 9 Department of Forensic Medicine, Medical University of Warsaw, Warsaw 02-007, Poland

## Abstract

The Neurofascins (NFASCs) are a family of proteins encoded by alternative transcripts of *NFASC* that cooperate in the assembly of the node of Ranvier in myelinated nerves. Differential expression of *NFASC* in neurons and glia presents a remarkable example of cell-type specific expression of protein isoforms with a common overall function. In mice there are three NFASC isoforms: Nfasc186 and Nfasc140, located in the axonal membrane at the node of Ranvier, and Nfasc155, a glial component of the paranodal axoglial junction. Nfasc186 and Nfasc155 are the major isoforms at mature nodes and paranodes, respectively. Conditional deletion of the glial isoform Nfasc155 in mice causes severe motor coordination defects and death at 16–17 days after birth. We describe a proband with severe congenital hypotonia, contractures of fingers and toes, and no reaction to touch or pain. Whole exome sequencing revealed a homozygous *NFASC* variant chr1:204953187-C>T (rs755160624). The variant creates a premature stop codon in 3 out of four *NFASC* human transcripts and is predicted to specifically eliminate Nfasc155 leaving neuronal Neurofascin intact. The selective absence of Nfasc155 and disruption of the paranodal junction was confirmed by an immunofluorescent study of skin biopsies from the patient versus control. We propose that the disease in our proband is the first reported example of genetic deficiency of glial Neurofascin isoforms in humans and that the severity of the condition reflects the importance of the Nfasc155 in forming paranodal axoglial junctions and in determining the structure and function of the node of Ranvier.

## Introduction

Neurofascin (NFASC) proteins are encoded by *NFASC*. They are members of the L1 family of immunoglobulin cell adhesion molecules and play a critical role in the assembly of the node of Ranvier, the axonal domain in myelinated nerves that is responsible for rapid nerve impulse conduction in the vertebrate central and peripheral nervous systems (CNS and PNS) (1–3). In mice there are three Neurofascin proteins however, Nfasc186 and Nfasc155 are the major isoforms responsible for node assembly (4–6).

Nfasc186 is located in the axonal membrane at the node of Ranvier in myelinated vertebrate nerves whereas Nfasc155 is a glial component of the paranodal axoglial junction that flanks the node (2,6). Nfasc155 is expressed by myelin-forming oligodendrocytes in the CNS and Schwann cells in the PNS. Both Neurofascin isoforms have each been shown to play crucial roles in the clustering of voltage-gated sodium (Nav) channels at the node of Ranvier (1–3). Although Nfasc186 is not essential for the clustering of Nav channels at the unmyelinated proximal domain of axons, the Axon Initial Segment (AIS), it is required for the long-term maintenance and stability of the AIS (7).

Interestingly, the clustering of Nav at the node of Ranvier can be accomplished either by Nfasc186, or by the formation of a paranodal junction by oligodendrocyte Nfasc155 with its axonal binding partners Caspr and Contactin (3,6). Nevertheless, even when Nfasc186 is present, mice specifically lacking the glial isoform Nfasc155 show severe motor coordination defects and die prematurely at 16–17 days after birth (8). Furthermore, specific loss of Nfasc155 has been shown to disrupt synapse elimination at the developing neuromuscular junction (9). This is surprising since disruption of the paranodal axoglial complex through the loss of Caspr is much less severe and mice live beyond 5 months (10). Taken together, these data from mouse studies show that loss of Nfasc155 has a severe phenotype beyond the simple disruption of the paranodal axoglial junction.

Recently, it has been reported that patients with chronic inflammatory demyelinating polyneuropathy possess anti-Nfasc155 immunoglobulin antibodies (11). Further, it has been proposed that a T-helper1 response against Nfasc186 may be used as a biomarker for multifocal acquired demyelinating sensory and motor polyneuropathy (12). However, thus far mutations in the *NFASC* gene as a cause of a human mendelian disorder have not been described.

In the present study we describe a proband with a novel homozygous *NFASC* mutation that is predicted to selectively affect Nfasc155 leaving the neuronal isoforms intact. We confirm the selective absence of oligodendrocyte Nfasc155 and disruption of the ternary complex at the paranodal junction. Moreover, and consistent with previous studies on mice, loss of Nfasc155 leads to a severe clinical phenotype.

## Results

**Figure 1:**
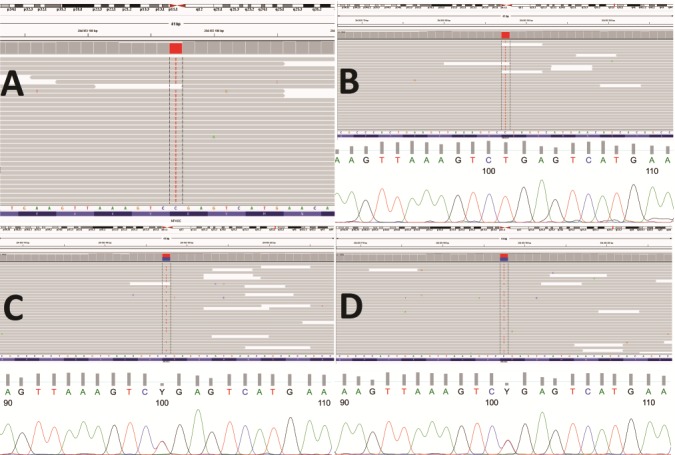
Results of genetic analyses of rs755160624 (NM_015090.3 c.2491C>T, (p.Arg831Ter) in the *NFASC* gene. (**A**) WES in proband (IGV view), (**B–D**) results of Sanger sequencing and ADS confirming the homozygosity of proband (B total count 11436, C-143 (1%), T-11207 (98%)), and demonstrating heterozygosity of her mother (C total count 9774, C-4823 (49%), T-4902 (50%)) and father (D total count 12620, C-6317 (50), T-6245 (49%)).

**Figure 2:**
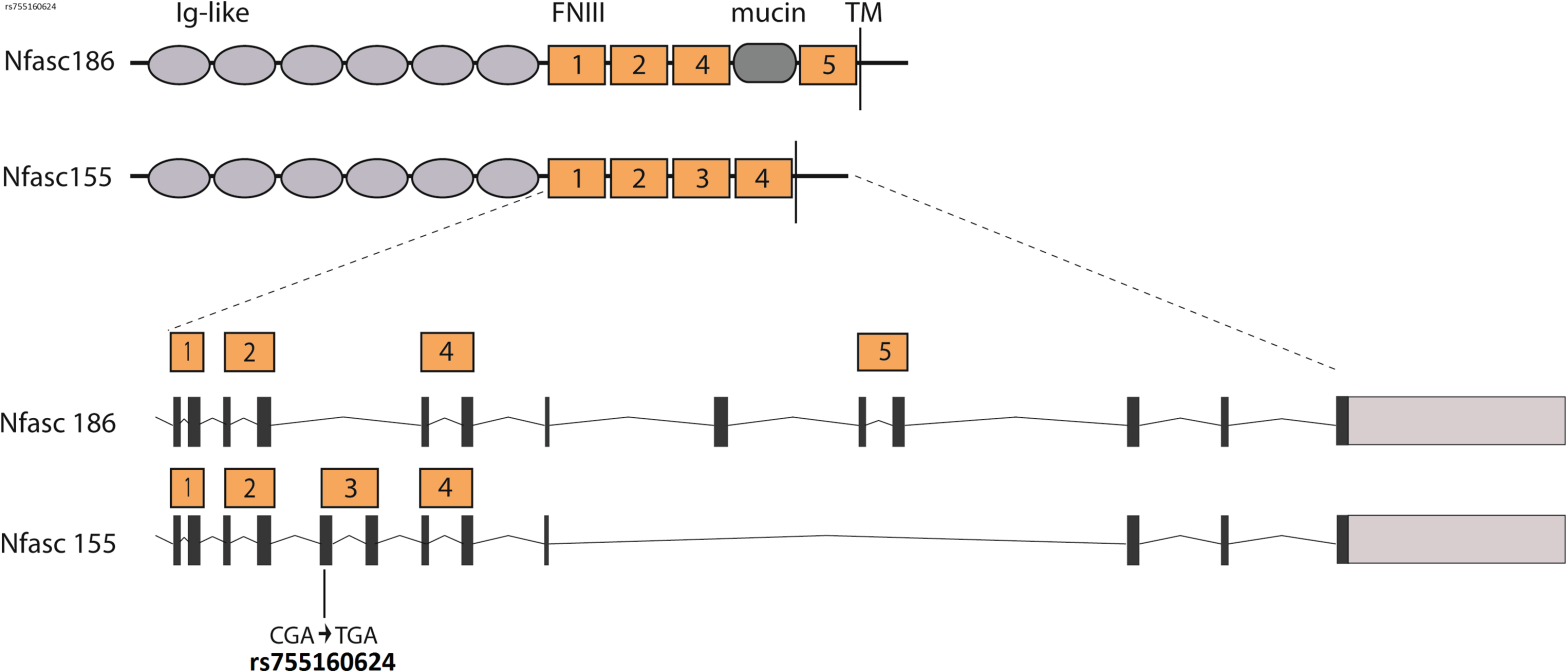
Schematic view of the domain structure of neuronal and glial human Neurofascin isoforms, Nfasc186 (NM_001005388.2) and Nfasc155 (NM_001160331.1), showing the exons that encode the five Fibronectin Type III (FNIII) repeats. The homozygous rs755160624 variant found in the proband creates a stop codon in the third FNIII repeat specific to the glial Nfasc155 isoform.

Bioinformatics analysis of whole exome sequencing (WES) data revealed a homozygous variant chr1:204953187-C>T (rs755160624) in the *NFASC* gene (Fig. 1). rs755160624 is predicted to create a premature stop codon in three out of the following four *NFASC* transcripts: NM_015090.3, c.2491C>T, (p.Arg831Ter), exon 21 of 27, amino acid (aa) 831 of 1170; NM_001160331.1, c.2536C>T, (p.Arg846Ter), exon 20 of 27, aa 846 of 1190 and NM_001160332.1, c.2491C>T, (p.Arg831Ter), exon 21 of 28, aa 831 of 1175 (RefSeq Transcripts Version: 27-Feb-2018). In the remaining *NFASC* transcript NM_001005388.2 the variant is predicted to affect a noncoding position located deeply within intron 21 (intronic position 2039 of 5397, c.2470 + 2039C>T). NM_001005388.2 is the canonical *NFASC* transcript encoding the longest protein isoform of 1241 aa corresponding to Nfasc186 (5). Thus, homozygosity for rs755160624 is predicted to cause loss of shorter noncanonical NFASC isoforms including the human counterpart of glial Nfasc155 (NM_001160331.1), while leaving the longest (neuronal) isoform intact (5) (Fig. 2). A family study using amplicon deep sequencing (ADS) and Sanger sequencing confirmed the homozygosity for rs755160624 in the proband and showed the heterozygous carrier status of her healthy parents (Fig. 1).

rs755160624 has been previously reported in four heterozygous subjects from the GnomAD database three of whom were from a European population and one from a Finnish population (European population frequency of 1 in 21114 subjects, http://gnomad.broadinstitute.org/). The rs755160624 was not found in an in-house database of >1000 Polish exomes.

Given the low population frequency of rs755160624 we reexamined the issue of the relatedness of proband’s parents. When interviewed again the parents confirmed lack of consanguinity but reported that the paternal grandmother of the proband’s mother was born in the same small village in Ukraine as the grandparents of the proband’s father. We also analyzed the WES data for runs of homozygosity (ROHs). In the proband we found four ROHs ranging from 5.37–2.65 Mb with a total length of 13.83 Mb. rs755160624 was located within one of the detected ROHs with a length of 3.04 Mb. Analysis of ROHs in 324 samples from unrelated subjects from a Polish population in which WES was performed with the same protocol as in the proband’s showed that in 28 (9%) of samples the summed ROH length was longer or equal than in proband.

### Immunofluorescence studies

**Figure 3:**
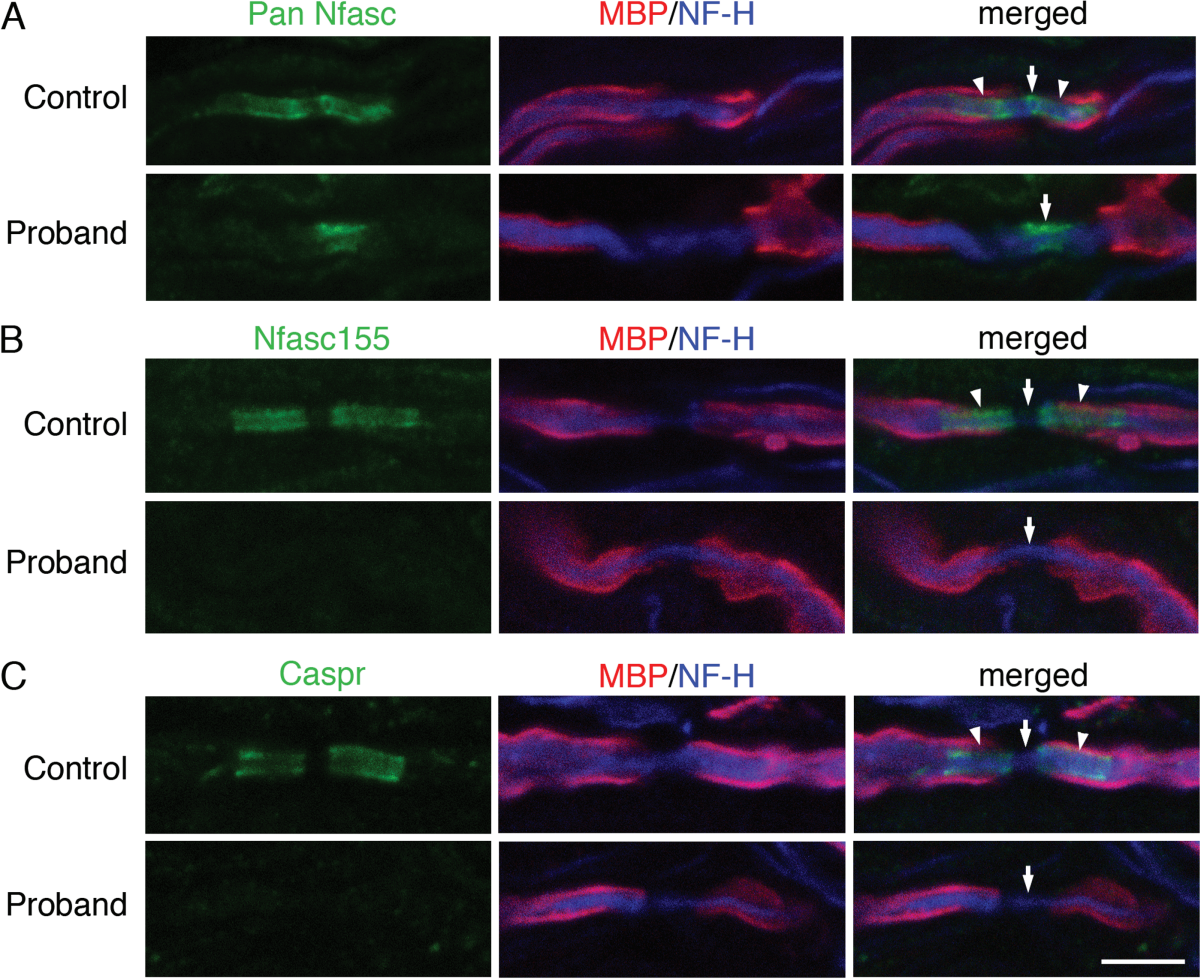
Nfasc155 is absent at the paranode of peripheral myelinated axons. Sections (5 mm thick) of biopsies from control and proband glabrous skin from infants of 11 and 2 months of age, respectively, were stained by immunofluorescence to reveal myelinated axons using antibodies against both MBP and NF-H. (**A**) The presence of both nodal neuronal NFASC isoforms (Nfasc186 and Nfasc140) at the node and glial Nfasc155 at the paranodal axoglial junction is revealed by staining with a pan-Nfasc antibody in the control. However, only the nodal isoforms are present in proband nerve fibres. The glial paranodal Nfasc155 is missing. (**B**) An antibody specific for glial Nfasc155 immunostains the paranodes of control but not proband nerve fibres, confirming the absence of this NFASC isoform in the proband. (**C**) Caspr is an axonal component of the paranodal axoglial complex with glial Nfasc155. Loss of Caspr at the paranodes in the proband confirms that the paranodal junction is disrupted due to the loss of Nfasc155. The arrowheads point to the location of paranodes and the arrows point to nodes. Size marker, 5 mm.

In order to confirm the predicted lack of Nfasc155 in nerves we evaluated peripheral myelinated axons in skin biopsies from the proband and control. Nfasc155 was absent at the paranode of myelinated axons in the proband (Fig. 3). Sections of biopsies from control and proband glabrous skin were stained by immunofluorescence to reveal myelinated axons using antibodies against both myelin basic protein (MBP) and neurofilament H (NF-H). The presence of both neuronal Neurofascin at the node and glial Nfasc155 at the paranodal axoglial junction in the control was revealed by staining with a pan-Nfasc antibody. However, only the nodal isoforms were present in proband nerve fibres while the glial paranodal Nfasc155 was missing (Fig. 3). Furthermore, an antibody that is specific for glial Nfasc155 immunostained the paranodes of control but not proband nerve fibres, confirming the absence of this Neurofascin isoform in the proband. Axonal Caspr and Contactin and glial Nfasc155 together form the ternary axoglial junctional complex at the paranode (Fig. 3). Hence, the loss of Caspr at the paranodes in the proband confirmed the disruption of the axoglial complex as a result of the absence of Nfasc155 in the proband (Fig. 3).

## Discussion

We found a recessive, homozygous *NFASC* variant rs755160624 in the proband which is associated with severe and progressive neurological disease. rs755160624 *NFASC* variant is located in the third FNIII repeat specific for the glial NFASC155 isoform (Fig. 2). In the homozygous state this *NFASC* variant is thus predicted to cause selective loss of the glial-specific Nfasc155 isoform whereas Nfasc186, the major neuronal isoform at the node of Ranvier, should not be affected. This prediction was substantiated by an immunofluorescence study using a pan reactive Nfasc antibody which demonstrated the lack of glial paranodal Nfasc155 in the proband, whereas neuronal NFASC was present. Furthermore, Caspr was absent from the paranodes of the proband confirming the disruption of the paranodal junction.

On clinical grounds proximal spinal muscular atrophy (SMA) was initially suspected in our proband. Symptoms supporting the SMA diagnosis included weak fetal movements, polyhydramnios, severe hypotonia, congenital contractures and primary respiratory distress. However, a lack of response to light and epilepsy indicating involvement of the CNS was not typical for SMA and we suggest that these symptoms could be useful for clinical differentiation between SMA and the *NFASC* associated disease.

The loss of nociceptive responses may reflect deficiencies in both the CNS and PNS. Myelinated A delta nerve fibers responsive to mechanical and painful stimuli normally have much slower conduction velocities than A beta fibers in peripheral nerves. From our mouse work we would predict a further 50% reduction in conduction velocities in these peripheral nerves as a result of the loss of intact paranodal axoglial junctions due to the absence of Nfasc155 (9). This would seem likely to contribute to decreased responses to mechanical and painful stimuli in the patient, although we cannot preclude the effect of reduced conduction velocities in the CNS as a further contributory factor.

Mice lacking all NFASC isoforms die at post-natal day 7 (P7) (1). NFASC-null mice that over-express either of the two neuronal isoforms (Nfasc186 or Nfasc140) survive into adulthood but are ataxic, presumably because they do not have intact paranodal axoglial junctions (no Nfasc155) in their myelinated nerves (2). These mice have conduction velocities in their peripheral nerves that are 50% of normal. However, selective deletion of glial Nfasc155 in the presence of normal levels of neuronal NFASC causes a very severe phenotype in mice resulting in early postnatal death (8,9). Clearly, over-expression of nodal NFASC can compensate for the disruption of the paranodal axoglial junction to a significant degree. However, why the absence of Nfasc155 should cause a much more severe phenotype in mice than loss of Caspr is still not understood. Nevertheless, the phenotype of our proband reflects the severity of the murine phenotype and confirms that the paranodal, glial isoform of NFASC, Nfasc155, has a particularly important role in nodal function.

Homozygosity for an extremely rare variant in a Polish population such as rs755160624 in a child of a reportedly non-consanguineous couple is unusual and raises the possibility of an unknown/undisclosed relatedness. The finding that rs755160624 was within a relatively long (>3 Mb) ROH together with high (∼90 percentile) summed length of ROHs detected in the proband’s exome as well as the information that three of her grand-grand-parents were born in the same village all indicate that a low but distinct degree of parental consanguinity contributed to the proband’s rs755160624 homozygosity.

In conclusion, we provide the first description of the proband with a homozygous *NFASC* mutation resulting in the absence of glial NFASC155.

## Materials and Methods

### Clinical report

The proband was the first child of a healthy non-consanguineous Polish couple. The family history was noncontributory. The girl was born at 34 weeks of gestation by cesarean section. The pregnancy was affected by polyhydramnios and weak fetal movements. Hence, prenatally, fetal esophageal atresia was suspected. The birth parameters were the following: weight 2180 g (10–50 percentile), occipitofrontal circumference 31 cm (50 percentile), Apgar score 1/2/4/4 points in 1, 3, 5 and 10 min. The newborn was treated by intubation and mechanical ventilation because of respiratory insufficiency. The newborn presented no muscle tension and reflexes. No congenital defects were noted. Ultrasound examination showed normal heart structure. Physical examination revealed severe hypotonia and contractures of finger in hands and feet. Neurological consultation at 5 days of life showed no reaction to touch or pain, narrow, symmetric pupils, no response to light and tongue in midsaggital location, no fasciculations. Amimia, muscular hypotonia and areflexia were noted as well as motor and oral automatisms were absent. Head circumference was 32 cm, anterior fontanelle 2x2 cm at the skull level. Brain and cervical spine magnetic resonance imaging did not reveal any pathology apart from discrete ischemic and hypoxic changes. Electroencephalography showed unspecific changes. Tandem mass spectrometry, gas chromatography–mass spectrometry assays and aminoacids profile in serum and cerebro-spinal fluid revealed no abnormalities; lactic acid, CPK and NH_3_ levels were in the normal range. Levels of C and S proteins and the level of immunoglobulin were in normal range. Ophthalmology examination did not show any abnormalities.

*SMN1* and *SMN2* genetic tests (MLPA 060-B2 SMA) were performed and yielded normal results (no deletion of exon 7 and 8 in the *SMN1* gene and deletion of one allele of *SMN2* gene). Because of feeding difficulties percutaneous endoscopic gastrostomy was performed. Subsequent neurological consultation at 12 weeks showed vestigial spontaneous motorics (weak movement of upper and lower limbs), amimia, no eye contact, hypotonia, symmetric, extensor dominance, areflexia and absence of motor and oral automatism. Chronic respiratory insufficiency and severe neurological status without improvement prompted tracheostomy. At the age of 3 months, the infant was admitted to a palliative care unit. Severe hypotonia was observed with very low touch reaction, without sucking reflex, without eye contact. At the age of 4 months epilepsy appeared and was treated with valproate.

### Genetic study

Venous blood samples were collected from all participants of the study (proband and parents). Written informed consent was obtained prior to genetic testing from the parents (the legal guardians of the proband). Ethics approval was granted by the Institutional Review Board of Warsaw Medical University.

WES was performed on the proband using SureSelect Human All Exon V5 (Agilent Technologies, Palo Alto, CA) on HiSeq 1500 (Illumina, San Diego, CA). Bioinformatics analysis of WES data was performed using a previously described pipeline (13). The mean depth of coverage in the sequenced sample was 78x, 91% of target sequence was covered min 20x and 97% min 10x. In order to confirm the results from WES and to verify the carrier state of the participants of the study ADS was performed using Nextera XT Library Preparation Kit (Illumina, San Diego, CA). Sequencing was carried out on HiSeq 1500. The following PCR primers were used for ADS: NFASC_F - 5′AGT GTT GAG TGG AGT GCA GGT 3′, NFASC_R – 5′ TCT TAT AGC CCG CAT TTG CT 3′. The same primers were used for Sanger sequencing.

ROHs were detected using the bcftools program (14) with variants detected by the GATK HaplotypeCaller algorithm (15) as input. Variants were called only within the target enrichment region. GnomAD database (16) was used as the source of reference allele frequencies and genetic maps were taken from the 1000 Genomes project (17).

### Immunofluorescence study

Skin biopsies in the proband were obtained from the glabrous skin of a first toe using a 2 mm punch and were fixed in cold Zamboni’s fixative for 4–5 h, followed by washes with 100 mM Sorenson’s phosphate buffer (pH 7.4). The control biopsy was obtained from the glabrous skin of a supernumerary toe of an 11-month-old infant who underwent surgery for polydactyly. The tissue was cryoprotected with 25% sucrose in 100 mM Sorenson’s phosphate buffer and frozen in OCT (CellPath). Sections (5 um) were cut on a cryostat (Leica) and collected on Superfrost Plus slides. Sections were blocked with 5% fish skin gelatin (Sigma), 0.2% Triton-X100 in Phosphate-buffered saline (PBS) for one hour, followed by incubation in primary antibodies overnight at room temperature. After PBS washes sections were incubated for 1.5 h with secondary antibodies. Primary antibodies were used at the following dilutions: rabbit Pan Nfasc (NFC1) 1:2000 (6), rabbit Nfasc155 (NFF3) 1:1000 (6), rabbit Caspr 1:5000 (gift of Dr. D.R. Colman), rat MBP 1:200 (AbD Serotec) and Neurofilament-200 (Sigma) 1:1000. Secondary antibodies (Invitrogen Molecular Probes) were used at 1:1000 dilution and were goat anti-rabbit IgG AlexaFluor 488, goat anti-rat IgG AlexaFluor 594 and goat anti-mouse IgG1 AlexaFluor 647. Sections were mounted in Vectashield (Vector Laboratories). Confocal microscopy was performed using a Leica TCL-SL microscope with a 63x objective (1.4 NA).
